# Barriers to and strategies for early implementation of pharmacy-delivered HIV PrEP services in Kenya: An analysis of routine data

**DOI:** 10.3389/frph.2023.1023568

**Published:** 2023-02-21

**Authors:** Hilma N. Nakambale, Stephanie D. Roche, Peter Mogere, Victor Omollo, Alexandra P. Kuo, Andy Stergachis, Jared M. Baeten, Elizabeth Bukusi, Kenneth Ngure, Melissa Latigo Mugambi, Katrina F. Ortblad

**Affiliations:** ^1^ Department of Global Health, University of Washington, Seattle, WA, United States; ^2^ Public Health Sciences Division, Fred Hutchinson Cancer Center, Seattle, WA, United States; ^3^ Partners in Health Research and Development, Center for Clinical Research, Kenya Medical Research Institute, Nairobi, Kenya; ^4^ Centre for Microbiology Research, Kenya Medical Research Institute, Nairobi, Kenya; ^5^ Department of Pharmacy, University of Washington, Seattle, WA, United States; ^6^ Department of Epidemiology, University of Washington, Seattle, WA, United States; ^7^ Department of Medicine, University of Washington, Seattle, WA, United States; ^8^ Gilead Sciences, Foster City, CA, United States; ^9^ Department of Obstetrics and Gynecology, University of Washington, Seattle, WA, United States; ^10^ Department of Community Health, Jomo Kenyatta University of Agriculture and Technology, Nairobi, Kenya

**Keywords:** private pharmacies, PrEP, differentiated service delivery, implementation science, HIV prevention

## Abstract

**Background:**

For individuals who face challenges accessing clinic-based HIV pre-exposure prophylaxis (PrEP), differentiated service delivery models are needed to expand access and reach. During a pilot study testing a novel pharmacy-delivered oral PrEP model in Kenya, we used routine programmatic data to identify early implementation barriers and actions that providers and study staff took in response to the barriers.

**Methods:**

We trained pharmacy providers at five private pharmacies in Kisumu and Kiambu Counties to initiate and continue clients at risk of HIV acquisition on PrEP for a fee of 300 KES per visit (∼$3 USD) using a prescribing checklist with remote clinician oversight. Research assistants stationed at the pharmacies completed weekly observation reports of pharmacy-delivered PrEP services using a structured template. We analyzed reports from the first 6 month of implementation using content analysis and identified multi-level early implementation barriers and actions taken to address these. We then organized the identified barriers and actions according to the Consolidated Framework for Implementation Research (CFIR).

**Results:**

From November 2020 to May 2021, research assistants completed 74 observation reports (∼18/pharmacy). During this period, pharmacy providers screened 496 potential PrEP clients, identified 425 as eligible for pharmacy-delivered PrEP services, and initiated 230 (54%) on PrEP; 125 of 197 (63%) clients eligible for PrEP continuation refilled PrEP. We identified the following early implementation barriers to pharmacy-delivered PrEP services (by CFIR domain): high costs to clients (intervention characteristics), client discomfort discussing sexual behaviors and HIV testing with providers (outer setting), provider frustrations that PrEP delivery was time-consuming and disruptive to their workflow (inner setting), and provider hesitancy to deliver PrEP due to concerns about encouraging sexual promiscuity (characteristics of individuals). To help address these, pharmacy providers implemented a self-screening option for behavioral HIV risk assessment for prospective PrEP clients, allowed flexible appointment scheduling, and conducted pharmacy PrEP trainings for newly hired staff.

**Conclusion:**

Our study provides insight into early barriers to implementing pharmacy-delivered PrEP services in Kenya and potential actions to mitigate these barriers. It also demonstrates how routine programmatic data can be used to understand the early implementation process.

## 1. Introduction

HIV clinics at public healthcare facilities are the mainstay of HIV prevention in Kenya and provide oral pre-exposure prophylaxis (PrEP) for individuals at risk of HIV acquisition (1, 2). Barriers to clinic-based PrEP delivery, such as long wait times and HIV-associated stigma, continue to limit the reach of PrEP services (2, 3). In Kenya and many other low- and middle-income countries, private pharmacies are often the first place many individuals go to address their healthcare needs (4–6). These pharmacies already provide a variety of sexual and reproductive health (SRH) services and products, such as contraceptive methods (e.g., condoms and oral contraceptives) and treatment for sexually transmitted infections (STIs), which could be paired with the delivery of PrEP services (7). Qualitative research with pharmacy providers and clients in Kenya identified many potential perceived advantages of pharmacy-delivered PrEP services, including increased convivence, privacy, and shorter wait times (8).

A model of pharmacy-based PrEP delivery that is integrated with the delivery of SRH services could potentially address barriers to clinic-delivered PrEP services and provide clients with more comprehensive SRH services in this setting. In January 2020, we collaborated with Kenyan stakeholders from PrEP regulatory, professional, service delivery, civil society, and research organizations to design a model of pharmacy-delivered PrEP services based on ones currently ongoing in high-income settings (1, 9). In our model, pharmacy providers use a prescribing checklist to identify clients without any medical conditions that might contraindicate PrEP safety and dispense PrEP with remote clinician oversight, referring clients that do not meet the checklist's eligibility criteria to free PrEP services delivered by clinicians at nearby public clinics (1).

Systematically documenting the early implementation process of a new delivery model is an essential part of formative evaluation, which provides valuable insights into the complexity of implementation projects and helps answer questions about context, adaptations, and response to changes that can guide future implementation efforts (9, 10). For example, documenting implementation activities provides information for implementers, key evaluation stakeholders, and individuals who wish to implement similar interventions and can help inform how they might adapt or modify support activities to enhance implementation (11). Routinely collected programmatic data is increasingly being recognized as an important data source in implementation science that can capture the implementation process and help identify early implementation barriers and actions taken to address these (12, 13). Compared to participant-level research data, it is often collected more frequently throughout implementation, is less expensive to collect, and can capture more system-level implementation barriers and strategies. In this paper, we used such data to identify early implementation barriers to this novel model of pharmacy-based PrEP delivery in Kenya and actions that pharmacy providers and study staff took to help address these during the first 6 months of implementation.

## 2. Methods

### 2.1. Study setting and pharmacies

In Kenya, there are >3,000 private pharmacies licensed with the Kenya Pharmacy & Poisons Board (PPB) (14), including wholesale, hospital, and private community pharmacies (which count for the vast majority). The Pharmacy PrEP Pilot study (ClinicalTrials.gov: NCT04558554) took place at five privately-owned, community-based pharmacies; two in Kisumu County and three Kiambu County. Kisumu County is located in western Kenya and has a generalized HIV epidemic with a population-level prevalence of ∼17.5% (15). Kiambu County is located in central Kenya and has a concentrated HIV epidemic with a population-level prevalence of ∼4% (16). In 2022, there were 153 licensed pharmacies in Kisumu County (7 wholesale, 27 hospital, and 119 private community pharmacies) and 578 licensed pharmacies in Kiambu County (15 wholesale, 47 hospital, and 517 private community pharmacies) (14).

We purposely selected pharmacies for pilot participation that were interested in and willing to deliver PrEP services, served 100 to 300 clients per week, and were in settings (e.g., universities, bars, market areas) where the surrounding populations and clientele may be likely to engage in activities associated with HIV risk. Additionally, to be eligible for participation, pharmacies had to be licensed with the Kenya PPB and operated by pharmacists or pharmaceutical technologists, have a private room for confidential HIV testing and counseling (which are common among licensed pharmacies), and be willing to participate in training on our care pathway for pharmacy-delivered PrEP services. We did not engage pharmacies not licensed by the PPB for this research because PrEP service delivery in this community setting will likely require some governmental oversight in Kenya; especially if the PrEP drugs and HIV tests used to support delivery are provided by the Ministry of Health, as they were in this research study.

### 2.2. Pharmacy provider training and support

We trained pharmacy providers to broach the topic of PrEP with clients seeking SRH services associated with risk of HIV acquisition (e.g., condoms and emergency contraception) and to assess interested clients' eligibility for PrEP initiation or refills using a prescription prescribing checklist (Appendix I). The prescribing checklist outlines the core components of pharmacy-delivered PrEP services, including counseling on HIV risk and PrEP eligibility, screening for medical conditions that might contraindicate PrEP safety (e.g., kidney disease), testing for HIV (using provider-assisted HIV self-testing), and prescribing and dispensing PrEP (1). In cases where pharmacy providers had questions or needed clarification on patient eligibility, a remote clinician was available for consultation *via* phone call, a secure WhatsApp group, and text message.

### 2.3. Pharmacy PrEP clients

Individuals who met the following eligibility criteria were eligible to enroll in the study: age ≥18 years old, self-reported behaviors associated with risk of HIV acquisition [according to Kenya's Risk Assessment Screening Tool (RAST) (17)], confirmed HIV-negative status, no medical conditions that might contraindicate PrEP safety (e.g., no history of liver or kidney disease), and willing to participate in research activities and give written informed consent.

Clients paid a service delivery fee of 300 Kenyan Shillings (KES) [∼$3 United States Dollars (USD)] to pharmacy providers for each study visit; an amount we determined in collaboration with the pilot pharmacy providers to incentivize them to complete client counseling, HIV testing, and PrEP dispensing. Pilot pharmacies additionally received 5,000 KES (∼$50 USD) per month for documenting PrEP services rendered, storing study commodities, completing required reports, and allowing a research assistant to be stationed on site.

Our study protocol was reviewed and approved by the Scientific Ethics Review Unit at the Kenya Medical Research Institute (KEMRI/SERU/CMR/P00137/4042 & KEMRI/SERU/CCR/0175/4017) and the Human Subjects Division at the University of Washington (STUDY00009587). All participants completed written informed consent and were compensated 500 KES (∼$5 USD) for completing research activities (e.g., questionnaires); an amount equivalent to other PrEP implementation projects led by this study team.

### 2.4. Data collection

Experienced Kenyan research assistants were stationed full time at each of the study pharmacies to observe implementation of pharmacy-delivered PrEP services and complete questionnaires with clients following each pharmacy PrEP visit. Our main data source for this analysis were routinely collected observation reports, which the research assistants completed weekly during implementation. These structured reports (see Appendix II for the report template) asked the researchers to: (1) describe the characteristics of pharmacy clients most interested in pharmacy PrEP services, (2) report common questions and concerns participants had about these services, (3) describe any challenges providers encountered while delivering PrEP, and (4) identify any practices the pharmacies implemented to facilitate PrEP delivery. We also used data from participant questionnaires completed at each pharmacy visit to describe demographic characteristics (e.g., age, sex, education) of the pharmacy clients initiating PrEP services; more details on these participants, their utilization of pharmacy PrEP services, and their experiences with the intervention are reported elsewhere (18–20).

### 2.5. Analysis

We analyzed all routine observational reports completed during the first 6 months of implementation using a combination of inductive and deductive approaches. One author (HNN) first read through all of the reports, created a codebook of barriers and actions taken, and coded the reports. Three authors (AK, KFO, and MLM) reviewed 25% of the coded reports each and identified additional codes to add; author HNN applied the updated codebook to all reports.

To better understand the determinants of early implementation of pharmacy-based PrEP delivery, we then organized our findings according to the Consolidated Framework for Implementation Research (CFIR) (21). The CFIR contains 39 constructs hypothesized to influence implementation of an intervention that are organized into five domains: (1) intervention characteristics, (2) individual characteristics (i.e., characteristics of the individuals implementing or being targeted by the intervention), (3) inner setting (i.e., the organization in which implementation occurs), (4) outer setting (i.e., the community or system), and (5) implementation process (i.e., how implementation is executed) (8, 21, 22). We also identified the level (i.e., client, provider, and/or pharmacy) at which the different implementation barriers and response actions occurred.

Lastly, we conducted member checking (23) with pharmacy providers (*n* = 2), research assistants (*n* = 7), study coordinators (*n* = 2), and principal investigators (*n* = 3) involved in the pilot study to confirm if our preliminary findings were consistent with their experiences and identify any additional barriers and actions that were not captured in the weekly reports. All coding was conducted in Dedoose (v9.0.17, Los Angeles, USA).

## 3. Results

From November 2020 to May 2021, research assistants stationed at five pilot pharmacies completed 74 observation reports (∼18 reports/pharmacy). During the first 6 months of pilot implementation, pharmacy providers screened 496 potential PrEP clients, identified 425 as eligible for pharmacy-delivered PrEP services, and initiated 230 (54%) on PrEP; among clients eligible for a refill visit at 6 months, 63% (125/197) refilled PrEP. Among the clients that initiated PrEP, less than half (43%, 98/230) were <25 years old, roughly half were men (48%, 111/230), and only two (1%) previously used PrEP. The primary outcomes from the Pharmacy PrEP pilot (e.g., PrEP initiation and continuation) are published elsewhere (24). In April 2021, 5 months into pilot implementation, one pharmacy dropped out of the study because the pharmacy owner did not want their pharmacy to be associated with HIV service delivery; this pharmacy was replaced with a new pharmacy that same month.

Below, we describe barriers to implementing pharmacy-delivered PrEP services (organized by CFIR domain and construct) and the actions taken by pharmacy providers and study staff in effort to mitigate these barriers. Table 1 summarizes our findings and features illustrative quotes.

**Table 1:** Early implementation barriers to pharmacy-delivered PrEP services and actions taken in response.

CFIR constructs	Implementation barrier	Actions taken (in response to barrier)
**Intervention characteristics**		
Cost	**Clients:** • Some clients felt that the fee for PrEP services (300 KES) was too expensive. *[Reported at all pharmacies]* *“In [this] pharmacy, most people do not want to pay anything [for PrEP] and only [agree to] pay as they are getting reimbursed [for participating in the study].”*—*Notes from member checking*^a^	• Some providers encouraged clients to use the compensation they were receiving for pilot participation (500 KSH) to cover the cost of PrEP services.
**Outer setting**		
Patient needs and resources	**Clients:** • Some clients did not feel comfortable discussing their sexual behaviors with pharmacy providers. *[Reported at all pharmacies]* *“Some couples who come to buy condoms, when talked to about PrEP by the provider, fear talking about their risks when together.”—Weekly report*^b^ *“Some clients aren't ready to talk about their sexual behaviors.”—Weekly report*^b^ • Some clients are hesitant to test for HIV for fear of receiving a positive result. *[Reported at all pharmacies]* *“Some pharmacy clients want PrEP and don’t want to be tested [for HIV].”—Weekly report*^b^	• Pharmacy providers reassured clients of the confidential nature of their interactions. • Some pharmacies (Pharmacies B and C) let clients complete the HIV risk assessment tool on a tablet (vs. verbally over the counter) and only probed further about their sexual behaviors if necessary. • During discussions with prospective PrEP clients, providers explained why HIV testing is an important and required step for PrEP initiation. • Providers also reassured clients that HIV testing would be conducted in a private room with results kept confidential and encouraged them to come back if/when they felt ready. *“[Pharmacy providers] Reassuring participants of privacy and confidentiality”—Weekly report*^b^
**Inner setting**		
Compatibility	**Providers:** • Some providers found initiating clients on PrEP to be time-consuming and disruptive to their workflow. *[Reported at all pharmacies]* *“The [PrEP] initiation process takes too long, and [pharmacy] providers have to attend to other clients.”—Notes from member checking*^a^	• Some providers asked PrEP clients to come back at a time when they anticipated the pharmacy would be less busy and they would have ample time to serve the client. • Some providers multi-tasked, completing other PrEP-related tasks and/or serving other clients during natural breaks in the PrEP delivery process (e.g., while waiting ∼20 min for HIV test results). *“The pharmacy provider fills [out] the prescriber’s checklist with the [study] participant as the HIV test still runs to save on time, especially for participants being enrolled who are in a bit of a hurry.”—Weekly report*^b^
Available resources	**Pharmacies:** • Provider turnover was high at some pharmacies and newly hired providers were not familiar with PrEP pharmacies. *[Reported at Pharmacies D]* *“High pharmacy provider turnover [is a challenge]. New ones come [i.e., start working at the pharmacy] and don't know how to initiate PrEP [or] identify clients [i.e., potential PrEP clients].”—Weekly report*^b^	• Study staff held trainings whenever needed to ensure newly hired pharmacy providers had the requisite knowledge and skills to deliver PrEP.
**Characteristics of individuals**		
Knowledge and beliefs about the intervention	**Providers:** • Some providers were hesitant to deliver PrEP out of concern that it would encourage clients to be promiscuous. *[Reported at Pharmacies A and D]* *“Pharmacy provider is not confident to engage on a PrEP talk.”—Weekly report*^b^	• Study staff held trainings whenever needed to ensure newly hired pharmacy providers had the requisite knowledge and skills to deliver PrEP.

^a^ These notes were recorded by research staff during our meeting with pharmacy providers and research staff that participated in implementation.

^b^ These weekly reports were completed by research assistants stationed at the pharmacy.

### 3.1. Intervention characteristics

#### 3.1.1. Cost

According to research assistants and pharmacy providers, many clients indicated that the price pharmacies were charging for PrEP services (300 KES) was too high. Some clients expressed a desire for the pharmacy to offer PrEP for free—as is done in public health facilities—or at a lower price, as this would make it easier for them to afford obtaining PrEP from the pharmacy. To mitigate this barrier, providers reminded clients that they would receive 500 KES compensation for participating in research activities (e.g., questionnaires) and so, in a sense, were receiving PrEP “for free”. According to pharmacy providers, this reframing resulted in some clients initiating PrEP despite the service fee.

### 3.2. Outer setting

#### 3.2.1. Patient needs and resources

Pharmacy providers and research assistants noted that some prospective PrEP clients were uncomfortable discussing their sexual behaviors during HIV risk assessment and/or were hesitant to test for HIV at the pharmacy for fear of testing positive. In response, some providers gave clients the option to screen themselves for HIV risk, allowing them to read the HIV risk assessment questions (e.g., “In the past six months, have you had sex with more than one partner?”) on an electronic tablet and mark down their answers. Providers also helped ease fears related to HIV testing by reassuring clients that they would conduct the HIV testing in a private room and keep their test results confidential.

### 3.3. Inner setting

#### 3.3.1. Compatibility

Reports indicated that delivering PrEP generally took providers more time than other services they offer and that backlogs in duties were not uncommon, especially when only a single pharmacy provider was on shift. Providers were especially concerned when other pharmacy clients were kept waiting while they attended to PrEP clients, and research assistants observed that this sometimes led the provider to rush through parts of PrEP delivery, such as counseling: *“Some providers missed discussing all the details [of PrEP] due to other consumers [i.e., pharmacy clients waiting to be seen]”* (pharmacy provider in member-checking meeting held in June 2021).

To address this issue, some pharmacy providers asked PrEP clients to return to the pharmacy during times of the day when the pharmacy was less busy. Additionally, some providers delivered parts of the intervention concurrently, such as completing the prescribing checklist during the 20-min wait for the HIV self-test to process.

#### 3.3.2. Available resources

At one pharmacy, high turnover of pharmacy staff hindered implementation of PrEP delivery. To ensure service continuity, study staff and pharmacy providers held on-the-job trainings, as needed, to ensure newly hired pharmacy providers had the requisite knowledge and skills to deliver PrEP.

### 3.4. Characteristics of individuals

#### 3.4.1. Knowledge and beliefs about the interventions

A few providers were not comfortable providing PrEP because they believed it would encourage clients to be more sexually “promiscuous.” In response, study staff encouraged these pharmacy providers to take a non-judgmental approach when serving PrEP clients and reminded them that the goal of PrEP is to protect clients from HIV, regardless of whether they—the pharmacy providers—morally agree with clients' sexual behaviors.

## 4. Discussion

In the early implementation phase of a novel pharmacy-delivered PrEP model in Kenya, we used routine programmatic data to identify several barriers and actions taken by pharmacy providers, with the support of study staff when needed, to improve implementation. Specifically, we identified barriers to pharmacy-delivered PrEP services across multiple CFIR domains, including characteristics of the intervention (i.e., high intervention cost to clients), the outer and inner settings (i.e., client discomfort discussing sexual behaviors with providers; time-consuming service delivery and workflow disruption for providers), and characteristics of the individuals implementing the intervention (e.g., provider hesitance to deliver PrEP because of concerns it would encourage behaviors associated with HIV risk). We also identified actions taken by pharmacy providers to help address these barriers, including implementation of an HIV risk self-screening option for prospective clients, flexible appointment scheduling, and re-training sessions for newly hired providers. While one pharmacy dropped out of the study during in this early implementation phase, this did not impact on our findings, which were consistent across all the participating pharmacies. In this study, we additionally demonstrated how routine programmatic data can be used to better understand the early implementation process.

Delivering high-quality PrEP services at private pharmacies may require more time than other pharmacy-delivered services. This may be particularly true during the early implementation phase, when pharmacy providers are new to the delivery of PrEP services, including screening for HIV risk, counseling on PrEP safety, and HIV testing. During the pilot, pharmacy providers served prospective PrEP clients on a walk-in basis, which is standard practice for private pharmacies in Kenya. However, the time it took to serve PrEP clients often disrupted providers' normal workflow and sometimes prevented them from meeting their daily delivery targets for other products. Future implementation efforts may consider instituting a flexible scheduling option for PrEP initiation and refill visits so that pharmacy providers can see PrEP clients during times that are typically less busy; which may help prevent workflow disruptions and increase the feasibility and sustainability of the intervention (25).

Stigma related to HIV treatment and prevention interventions from clients, providers, or the community can pose significant barriers to pharmacy-delivered PrEP services (8). During the pilot, some clients were hesitant to discuss their sexual behavior with pharmacy providers due to fear of judgment and some providers were uncomfortable talking to clients about PrEP because of personal concerns that PrEP use may increase clients' behaviors associated with HIV risk. Additionally, one pharmacy dropped out of the pilot because of concerns that the community would label their pharmacy as a place serving individuals living with HIV and that this would result in lost business. To facilitate the effective delivery of pharmacy-delivered PrEP services, pharmacy providers need to be equipped with knowledge through continuous high-quality training, including training on HIV stigma reduction (26) and the positive health outcomes that can be achieved with consistent PrEP use. Additionally, pharmacy-delivered PrEP services could potentially benefit from enabling certain components (e.g., HIV risk assessment) to be delivered remotely, as telehealth approaches have been found to reduce stigma for clients obtaining HIV services in other settings (27).

The cost of pharmacy-delivered PrEP services is a critical determinant of PrEP accessibility in this setting. Some clients in the pilot study expressed that they would not, in the long term, be able to afford a service fee of 300 KES for pharmacy-delivered PrEP services, and a few providers indicated that they would need to charge more than 300 KES to make the delivery of PrEP services worth their time. These findings highlight that for pharmacy-delivered PrEP services to be self-sustaining, the delivery of these services need to be profitable to pharmacies as well as affordable to clients (27–29). Across Kenya, private pharmacies vary considerably in size, service offerings, operating costs, and the average socioeconomic status of their clientele. Thus, a “one-fee-fits-all” approach may be unlikely to work and future research is needed to test different cost structures. Additionally, public-private partnerships may be needed to make pharmacy-delivered PrEP services affordable to populations of interest that might benefit most. For example, the Ministry of Health could donate PrEP drugs and HIV tests to pharmacies and pharmacies could then deliver services for a reduced service fee, paid by clients (like in this pilot), a public-sector payer, an international donor, or some combination thereof.

Our study has some limitations. First, because the study focused on early-stage implementation barriers, it did not capture mid- and late-stage implementation barriers that likely differ and may require additional implementation strategies to address. Second, this study relied on routine reports compiled by research assistants; as such, it likely does not capture all relevant implementation barriers, particularly ones faced by clients. A more in-depth investigation of client experiences with pharmacy-delivered PrEP services is forthcoming in qualitative research conducted by this team. Third, because research assistants were stationed full time at the study pharmacies, this might have reduced barriers to pharmacy-delivered PrEP services because any implementation-related provider questions could be address in real time and providers might have prioritized the delivery of PrEP over other products while under observation. Future research studies should consider removing research assistants from the pharmacies and using routinely collected pharmacy records to measure clinical and implementation outcomes related to PrEP delivery. Finally, because this pilot only took place at five private pharmacies in two counties, our findings may not be generalizable to all private pharmacies in Kenya or other similar settings.

Our study contributes to the literature by systematically identifying critical, early-stage barriers to implementing pharmacy-delivered PrEP services in Kenya. From this study, we gained insight into factors that can challenge PrEP delivery in this setting, such as cost, demands on provider time, and stigma related to HIV prevention services. Future implementers of pharmacy-delivered PrEP services should consider strategies such as allowing for flexible appointment scheduling, providing anti-bias training to providers, and implementing cost-sharing options. Future studies should assess barriers to implementation at different time points, incorporate input from other key stakeholders (especially clients), and measure the impact of strategies aimed to improve implementation of PrEP and SRH service uptake and continuation.

## Data Availability

The original contributions presented in the study are included in the article/**Supplementary Material**, further inquiries can be directed to the corresponding author.

## References

[1] Ortblad KF Mogere P Roche S Kamolloh K Odoyo J Irungu E Design of a care pathway for pharmacy-based PrEP delivery in Kenya: results from a collaborative stakeholder consultation. BMC Health Serv Res. (2020) 20(1):1–9. 10.1186/s12913-020-05898-9 PMC7661206 33176785

[2] Kenya - PrEPWatch. Available at: https://www.prepwatch.org/countries/kenya/ (Accessed August 7, 2022).

[3] At a glance: HIV in Kenya | be in the KNOW. Available at: https://www.beintheknow.org/understanding-hiv-epidemic/data/glance-hiv-kenya (Accessed August 7, 2022).

[4] Alshahrani AM Alsheikh MY. Community pharmacists’ perceptions, barriers, and willingness for offering sexual and reproductive health services. Int J Environ Res Public Health. (2021) 18(20):10735. 10.3390/ijerph182010735 34682503 PMC8535476

[5] Hernandez JH Mbadu MF Garcia M Glover A. The provision of emergency contraception in Kinshasa's private sector pharmacies: experiences of mystery clients. Contraception. (2018) 97(1):57–61. 10.1016/j.contraception.2017.08.001 28803883 PMC5745145

[6] Gonsalves L Wyss K Gichangi P Hilber AM. Pharmacists as youth-friendly service providers: documenting condom and emergency contraception dispensing in Kenya. Int J Public Health. (2020) 65(4):487–96. 10.1007/s00038-020-01348-9 32472373 PMC7275003

[7] Coroon M Kebede E Spektor G Speizer I. Key role of drug shops and pharmacies for family. Glob Heal Sci Pract. (2016) 4(4):594–609. 10.9745/GHSP-D-16-00197 PMC5199177 28031299

[8] Roche SD Wairimu N Mogere P Kamolloh K Odoyo J Kwena ZA Acceptability and feasibility of pharmacy-based delivery of pre-exposure prophylaxis in Kenya: a qualitative study of client and provider perspectives. AIDS Behav. (2021) 25(12):3871–82. 10.1007/s10461-021-03229-5 33826022 PMC8602157

[9] Tung EL Thomas A Eichner A Shalit P. Implementation of a community pharmacy-based pre-exposure prophylaxis service: a novel model for pre-exposure prophylaxis care. Sex Health. (2018) 15(6):556–61. 10.1071/SH18084 30401342

[10] Stetler CB Legro MW Wallace CM Bowman C Guihan M Hagedorn H The role of formative evaluation in implementation research and the QUERI experience. J Gen Intern Med. (2006) 21(Suppl. 2):1–8. 10.1007/s11606-006-0267-9 16637954 PMC2557128

[11] Keith RE Crosson JC O’Malley AS Cromp DA Taylor EF. Using the consolidated framework for implementation research (CFIR) to produce actionable findings: a rapid-cycle evaluation approach to improving implementation. Implement Sci. (2017) 12(1):1–12. 10.1186/s13012-017-0550-7 28187747 PMC5303301

[12] Thomas D Mujugira A Ortblad K Namanda S Kibuuka J Nakitende M A pragmatic approach to identifying implementation barriers and facilitators for a novel pre-exposure prophylaxis (PrEP) delivery model at public facilities in urban Uganda. Implement Sci Commun. (2022) 3(1):1–11. 10.1186/s43058-022-00254-w 35090560 PMC8795935

[13] Ortblad KF Mawandia S Bakae O Tau L Grande M Mogomotsi GP Using routine programmatic data to measure HIV incidence among pregnant women in Botswana. Popul Health Metr. (2022) 20(1):1–7. 10.1186/s12963-022-00287-2 35246143 PMC8896233

[14] Medpages statistics – pharmaceutical (pharmacies, distributors, etc.) in Kenya. Available at: https://www.medpages.info/sf/index.php?page=categorystats&countryid=23&categoryid=9 (Accessed December 21, 2022).

[15] NASCOP, MoH. Kenya population-based HIV impact assessment 2018-2019 preliminary report. Nascop (2020): p. 1–40. Available at: https://phia.icap.columbia.edu/countries/kenya/.

[16] National AIDS Control Council NACC. Ministry of health national AIDS control council. Ministry Health (2018). Available at: https//www.lvcthealth.org/wp-content/uploads/2018/11/KARPR-Report_2018.pdf, www.nacc.or.ke, www.nacc.or.ke.

[17] NASCOP. Framework for the implementation of pre-exposure prophylaxis of HIV in Kenya. NASCOP (2017) 96: p. 1–84. Available at: https://www.prepwatch.org/wp-content/uploads/2017/05/Kenya_PrEP_Implementation_Framework.pdf.

[18] Omollo V Asewe M Mogere P Gakuo S Kuo A Odoyo J The fidelity of a pharmacy-based PrEP delivery model in Kenya: an unannounced standardized patient actor assessment. Poster presentation at AIDS 2022: 24th international AIDS conference; Montreal, Canada (2022).

[19] Mogere P Omollo V Kuo A Asewe M Gakuo S Odoyo J The acceptability of pharmacy-based HIV PrEP delivery among retail pharmacy clients in Kenya: findings from a pilot study. E-poster presentation at AIDS 2022: 24th international AIDS conference; Montreal, Canada (2022).

[20] Ortblad KF Mogere P Omollo V Kuo A Asewe M Gakuo S Pharmacy-based PrEP initiation and continuation in Kenya: findings from a pilot study. Poster presentation at CROI 2022: 22nd conference on retroviruses and opportunistic infections, virtual; (2022).

[21] King DK Shoup JA Raebel MA Anderson CB Wagner NM Ritzwoller DP Planning for implementation success using RE-AIM and CFIR frameworks: a qualitative study. Front Public Health. (2020) 8:59. 10.3389/fpubh.2020.00059 32195217 PMC7063029

[22] Nilsen P. Making sense of implementation theories, models and frameworks. Implement Sci. (2015) 10(1):1–13. 10.1186/s13012-015-0242-0 25895742 PMC4406164

[23] Rolfe DE Ramsden VR Banner D Graham ID. Using qualitative health research methods to improve patient and public involvement and engagement in research. Res Involv Engagem. (2018) 4(1):1–8. 10.1186/s40900-018-0129-8 30564459 PMC6293564

[24] Ortblad KF Mogere P Omollo V Kuo A Asewe M Gakuo S Pharmacy-based PrEP initiation and continuation in Kenya: findings from a pilot study. Poster presentation at CROI 2022: 22nd Conference on Retroviruses and Opportunistic Infections, Virtual, February 12-16, 2022. Abstract 00928.

[25] Hopkins R Josma D Morris J Klepser DG Young HN Crawford ND. Support and perceived barriers to implementing pre-exposure prophylaxis screening and dispensing in pharmacies: examining concordance between pharmacy technicians and pharmacists. J Am Pharm Assoc. (2021) 61(1):115–20. 10.1016/j.japh.2020.10.005 33214059

[26] Maloney KM Krakower DS Ziobro D Rosenberger JG Novak D Mayer KH. Culturally competent sexual healthcare as a prerequisite for obtaining preexposure prophylaxis: findings from a qualitative study. LGBT Health. (2017) 4(4):310–4. 10.1089/lgbt.2016.0068 28514200 PMC5564027

[27] Traynor K. Telepharmacy service brings HIV preventive to rural iowans. Am J Heal Pharm. (2019) 76(6):331–2. 10.1093/ajhp/zxz001 31361843

[28] Koester KA Saberi P Fuller SM Arnold EA Steward WT. Attitudes about community pharmacy access to HIV prevention medications in California. J Am Pharm Assoc. (2020) 60(6):e179–83. 10.1016/j.japh.2020.06.005 PMC7875193 32665097

[29] Lopez MI Grant RM Dong BJ. Community pharmacy delivered PrEP to STOP HIV transmission: an opportunity NOT to miss!. J Am Pharm Assoc. (2020) 60(4):e18–24. 10.1016/j.japh.2020.01.026 32165026

