# Supplementary material for: Barriers to and strategies for early implementation of pharmacy-delivered HIV PrEP services in Kenya: An analysis of routine data
Source: Front Reprod Health. 2023 Feb 21;5:1023568. doi: 10.3389/frph.2023.1023568 (PMC9989195; doi:10.3389/frph.2023.1023568)
Supplement: Supplementary file 1 [file Datasheet1.pdf]

Appendix I

Pharmacy PrEP: Prescribing checklist

Pharmacy: ______________________ Provider: _______________________ Qualifications: ______________ MFL code: ___ ___ ___ ___ ___

CLIENT PROFILE:

Unique client record number: ___ ___ ___ ___ ___ / ___ ___ ___ ___/ ___ ___ ___ ___ ___

Visit date: dd / mm / yyyy

Initiating PrEP: ☐ Yes ☐ No

Name: First __________________ Middle __________________ Last ___________________ Telephone no: _____________

Alien/National ID/passport/Birth Cert No: __________________ Came to pharmacy for:___________________________________

PrEP initiation ONLY:

Sex: ☐ Male ☐ Female Date of birth: dd / mm / yyyy Age (years): ____ ____ If age <19, attends school: ☐ Yes ☐ No

Marital status: ☐ Never married ☐ Cohabiting ☐ Married monogamous ☐ Married polygamous ☐ Separated/divorced ☐ Widowed

Population type: ☐ Gen Population ☐ Discordant couple ☐ Key Population (Specify) ☐ MSM ☐ MSW ☐ FSW ☐ PWID

[If transferred in:] PrEP start date: dd / mm / yyyy Regimen: ☐ TDF-FTC ☐ TDF ☐ TDF-3TC

Facility transferred from: ___________________ MFL code: __ __ __ __ __ County: _________________

PrEP SCREENING:

Behavioral risk assessment: *Mark all that apply (past 6 months)*

Sex partner(s) is HIV+ AND:	☐ Yes	☐ No
not on ART, or On ART <6 months, or suspected poor ART adherence, or detectable viral load, or couple trying to conceive	☐ Yes	☐ No
Sex partner(s) high risk & HIV status is unknown:	☐ Yes	☐ No
Has sex with >1 partner	☐ Yes	☐ No
Ongoing IPV/GBV	☐ Yes	☐ No
Transactional sex	☐ Yes	☐ No
Recent STI (past 6 months)	☐ Yes	☐ No
Recurrent use of post-exposure prophylaxis (PEP)	☐ Yes	☐ No
Recurrent sex under influence of alcohol/recreational drugs	☐ Yes	☐ No
Inconsistent or no condom use		
Injection drug use with shared needles and/or syringes		

→ ***At HIV risk; <u>CONTINUE</u> with PrEP initiation at pharmacy***

Counseling

Willing to start/continue PrEP:	☐ Yes	☐ No
Adherence counseling Done:	☐ Yes	☐ No
Side effect counseling Done:	☐ Yes	☐ No
Family planning counseling Done:	☐ Yes	☐ No

→ ***CONTINUE with PrEP initiation at pharmacy***

Medical safety assessment

Signs & symptoms of acute HIV Infection	☐ Yes	☐ No
Liver disease:	☐ Yes	☐ No
Kidney disease:	☐ Yes	☐ No
Diabetes:	☐ Yes	☐ No
Other: _________________	☐ Yes	☐ No
[If female:] Pregnant	☐ Yes	☐ No
[If female:] Breastfeeding:	☐ Yes	☐ No

→ ***DO NOT start PrEP; refer to remote clinician***

PrEP refills ONLY:

Reported PrEP side effects:	*Describe:* _______________	☐ Yes	☐ No
[if yes:] Side effects severe:	_______________	☐ Yes	☐ No

→ ***DO NOT start PrEP; refer to remote clinician***

HIV testing

HIV test result: ☐ Negative ☐ Positive ☐ Indeterminate [Retest]

[If retest:] HIV test result: ☐ Negative ☐ Positive ☐ Indeterminate

→ ***DO NOT start PrEP; refer to remote clinician***

PrEP DISPENSING:

Regimen: ☐ TDF-FTC ☐ TDF ☐ TDF-3TC

# of months prescribed: ___ ___

Date of initiation: dd / mm / yyyy

Next appointment date: dd / mm / yyyy

Provider initials: ________

PrEP Rx

Appendix II

WEEKLY ACTIVITY SUMMARY

PHARMACY DELIVERY TO EXPAND THE REACH OF PrEP IN KENYA: PILOT STUDY

Pilot Pharmacy: Report by: Dates:

Summary for the week:

Number of potential PrEP users identified by the pharmacy provider	
Number of clients initiated PrEP previous week	
Number of clients initiated PrEP this week	
Number of clients initiating PrEP	
Number of follow up visits	Expected for month 1:<br>No. seen for month 1:

**Component**	**RA Observation**
Describe the pharmacy clients that are most interested in/likely to uptake PrEP *(including services purchased at pharmacy)*<br>Customers who come in to buy and/or ask for the following:<br>• Condoms<br>• Sex enhancers/boosters<br>• Pregnancy test kits/pregnancy testing<br>• PEP and/or PrEP<br>• Oral contraception and/or Emergency contraception<br>• HIV testing and/or HIVST Kits<br>• STI treatment	
What are common questions/concerns participants have about PrEP?	
Any challenges encountered? Elaborate	
What practices/solutions has the pharmacy instituted to make delivery easier?	
Highlight the discussion on the week's work with the pharmacy provider	
**Remarks**	
